# Electronic 12-Hour Dietary Recall (e-12HR): Comparison of a Mobile Phone App for Dietary Intake Assessment With a Food Frequency Questionnaire and Four Dietary Records

**DOI:** 10.2196/10409

**Published:** 2018-06-15

**Authors:** Luis María Béjar, Óscar Adrián Reyes, María Dolores García-Perea

**Affiliations:** ^1^ Department of Preventive Medicine and Public Health School of Medicine University of Seville Seville Spain; ^2^ Mutua Balear Seville Spain; ^3^ Virgen Macarena University Hospital Seville Spain

**Keywords:** dietary assessment, food frequency questionnaire, 24-hour dietary recalls, dietary record, mobile phone app

## Abstract

**Background:**

One of the greatest challenges in nutritional epidemiology is improving upon traditional self-reporting methods for the assessment of habitual dietary intake.

**Objective:**

The aim of this study was to evaluate the *relative validity* of a new method known as the current-day dietary recall (or *current-day recall*), based on a smartphone app called 12-hour dietary recall, for determining the habitual intake of a series of key food and drink groups using a food frequency questionnaire (FFQ) and four dietary records as reference methods.

**Methods:**

University students over the age of 18 years recorded their consumption of certain groups of food and drink using 12-hour dietary recall for 28 consecutive days. During this 28-day period, they also completed four dietary records on randomly selected days. Once the monitoring period was over, subjects then completed an FFQ. The two methods were compared using the Spearman correlation coefficient (SCC), a cross-classification analysis, and weighted kappa.

**Results:**

A total of 87 participants completed the study (64% women, 56/87; 36% men, 31/87).

For e-12HR versus FFQ, for all food and drink groups, the average SCC was 0.70. Cross-classification analysis revealed that the average percentage of individuals classified in the *exact agreement* category was 51.5%; *exact agreement + adjacent* was 91.8%, and no participant (0%) was classified in the *extreme disagreement* category. The average weighted kappa was 0.51.

For e-12HR versus the four dietary records, for all food and drink groups, the average SCC was 0.63. Cross-classification analysis revealed that the average percentage of individuals classified in the *exact agreement* category was 47.1%; *exact agreement + adjacent* was 89.2%; and no participant (0%) was classified in the *extreme disagreement* category. The average weighted kappa was 0.47.

**Conclusions:**

Current-day recall, based on the 12-hour dietary recall app, was found to be in good agreement with the two reference methods (FFQ & four dietary records), demonstrating its potential usefulness for categorizing individuals according to their habitual dietary intake of certain food and drink groups.

## Introduction

Many epidemiological investigations and intervention studies do not require a complete picture of the habitual diet (or average long-term consumption) [1], as it can represent both an unnecessary workload for study participants and an avoidable waste of the scarce resources available for research [2]. The essential concept of these studies is to determine habitual consumption of specific key groups of food and drink (hereafter referred to as ‘food’) [3-9]. Classifying individuals according to categories of habitual consumption of specific food groups is sufficient for identifying potential nutritional deficiencies [10] and for evaluating the relationship between relative ranking and disease [5,8-14], and effectiveness of personalized methods that are implemented to promote changes in dietary patterns regarding selected food groups [1,5,8-10,12].

The three principal and traditional self-reporting methods for determining dietary intake can be classified as follows: (1) Short-term methods: dietary record (DR) and 24-hour recall. (2) Long-term methods: food frequency questionnaire (FFQ). These methods present significant and well-documented limitations [5,11,15-18]. (1) DRs that require weighing food require too much preparation time and create a large workload for the study participants, which can lead to deviations in normal food intake (especially an underestimation of quantities), as well as both low participation and completion rates [5,11,15-17,19,20]. 24-hour recalls require trained personnel and depend on the short-term memory of participants. Additionally, a proper picture of habitual diet using short-term methods requires methods to be repeated multiple times [16,19], which increases problems inherent in these procedures. (2) FFQs depend primarily on long-term memory of the interviewed subject, do not account for intrapersonal variation in recording the daily consumption of food during the study period, and do not allow a precise estimation of serving sizes of food consumed. Due to these limitations, results obtained by these inexact methods over both the short- and long-term could lead to inexact conclusions and incorrect decisions [18].

Newer alternatives for determining dietary intake include audio signal processing, inertial sensing, image processing, non-intrusive near-infrared scanning, and gesture recognition interfacing [21-24]. Some authors maintain that more research is needed to develop these and other tools that are more objective and precise and that resources should be invested to this end [18]. Until these alternatives are available, digital technologies for self-reporting methods can, and must, be developed and utilized [25], as an improvement in traditional self-reporting methods. This progress constitutes one of the most important challenges in the field of nutritional epidemiology [4,20].

For these reasons, the need is evident to develop better methods that can eventually replace current traditional self-reporting ones and provide better accuracy in measuring usual dietary intake of free-living individuals. This upgrade would represent a great boon not only to researchers [10] but also to society in general, considering the critical repercussions from both epidemiological investigations and interventional studies regarding the dietary intake of the population at large [25].

The objective of this study was to determine the *relative validity* of the new method known as current-day dietary recall (or *current-day recall*), based on a smartphone app called 12-hour dietary recall (e-12HR—previously known as e-Epidemiology [25]), utilizing reference methods, such as a semiquantitative FFQ and four estimated DRs, to verify the comparability of consumption data regarding a list of key food groups in the three methods.

## Methods

### Recruitment

Students of the Schools of Medicine and Pharmacy at the University of Seville (Andalusia, Spain, South of Europe) participated in this study. Two classes from each School were randomly selected and one member of the research team presented the project. Participant recruitment took place from January to December 2017. Participants were incorporated in the study progressively during the entire recruitment period so that every day of the week and every season of the year could be represented [26]. Participation in the study was incentivized with a raffle that included school materials valued at 250 euros.

### Study Sample

The study was presented to 219 students and of those, 26 were not interested, and 98 did not meet the inclusion criteria. Of the 95 individuals who signed the informed consent, 87 completed the study. Inclusion criteria: (1) Older than 18 years of age; (2) a student of the Schools of Medicine or Pharmacy (University of Seville); and (3) possesses a smartphone with internet access (3G/4G/Wi-Fi) and Android operating system.

### First Interview: Participant Data Collection

The 95 students who were interested and met the inclusion criteria were scheduled for a first personal interview. The procedure of the interview was as follows: (1) the same research team member that presented the project personally explained the research protocol in detail; (2) each participant signed the informed consent form; (3) each participant was assigned a personal alphanumeric code; (4) each participant filled out an initial questionnaire (on paper) with date of birth, date of the interview, gender, and school; (5) each participant downloaded the e-12HR app for his or her personal smartphone; (6) the same member of the research team personally explained to each participant how to use the app with a practical demonstration before written instructions were given to the participants [14,27] to be consulted later, if necessary; (7) the same research team member personally gave each student detailed instructions on how to complete the four estimated DRs and how to estimate serving sizes consumed using an explanatory pamphlet that was also given to participants [12,13].

The researcher also insisted that participants maintain their habitual diet throughout the study.

### Completing the 12-Hour Dietary Recall App

The e-12HR app was developed to record daily consumption of a list of 10 food groups. The list could not be too long to minimize the workload on participants as well as the research costs [28]. These food groups were selected as they are indicators of health/disease and are considered protective factors (fruit, vegetables, legumes or fish) or risk factors (soft drinks, commercial baked foods, and precooked meals) for chronic illnesses [4,12,29]. They also provide consumption patterns that range from almost every day for every inhabitant of the population to infrequently for the majority [4] (Textbox 1).

**Figure 1:**
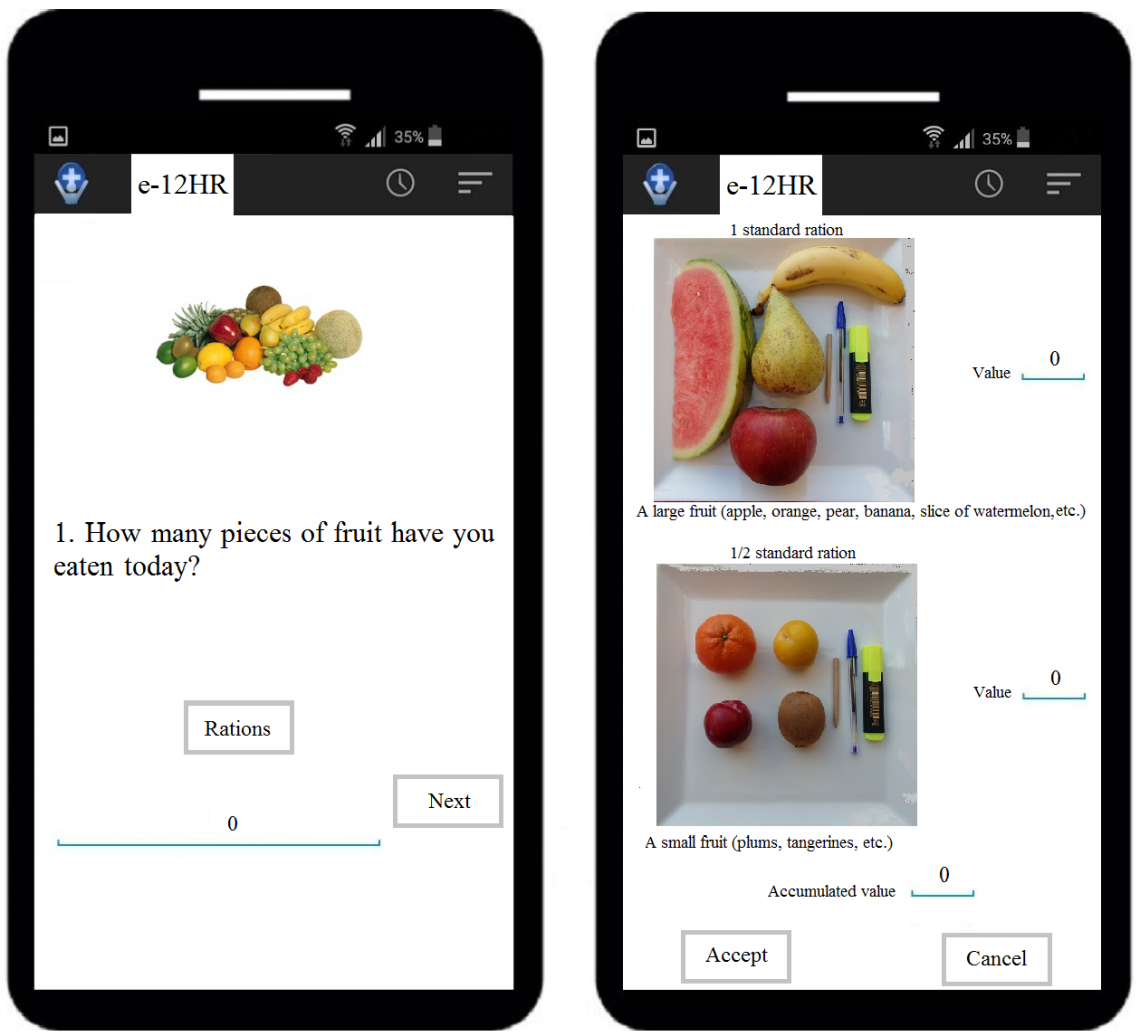
Screen capture of the 12-hour dietary recall (e-12HR).

The first time the e-12HR app was used, the participants were required to introduce their personally assigned alphanumeric code and the e-mail of the researcher who would receive the data from the app. Participants were instructed to complete the app after consuming the last food of the day [14,27]. The e-12HR app includes a notification option, so that the app can remind the participant, at the hour previously selected by the participant (between 20:00 h and 04:00 h), that it is time to complete the questionnaire. The app was structured according to food groups to facilitate completion of the e-12HR task. For each food group, the participant would choose the most appropriate image (or images) from a series of color photographs with 2-4 possible options, shown simultaneously [14,27], which illustrated the different serving sizes to assist with selecting the number of standard servings consumed [10,12,14,20,27]. To further assist with estimating serving sizes, each photograph was accompanied by explanatory text and three objects of known/predictable size for the students [30,31] (fiducial markers): a commonly used pencil, pen, and a marker. For example, on the first screen of the app, the following would appear: *How many pieces of fruit have you eaten today?*, with the *Rations* button, and *Next* button and a box with the value set to *0* by default. Supposing that the participant had, throughout the day, consumed an apple and three mandarins, they would proceed as follows: (1) tap the *Rations* button; (2) a new window opens with different photographs of fruit, an *Accept* button, and a *Cancel* button; (3) tap once on the photo corresponding to the apple and tap three times on the photograph corresponding to the mandarin; (4) tap the *Accept* button; (5) the app returns to the previous window but the box now has, instead of the *0* value, the corresponding number of standard rations for the fruit selected with the photographs, in this example, a value of *2.5* (1 × 1 standard ration + 3 × 0.5 standard ration); (6) tap the *Next* button to access the next food group and proceed as before; (7) if an error occurs, the participant can tap the *Cancel* button instead of Accept in step 4, starting the process over again (Figure 1).

The use of active images (accompanied by explanatory text and three reference objects of known/predictable size for the students [fiducial markers]) is the only modification of e-12HR regarding the previous version (known as e-Epidemiology). However, this single, apparently simple, modification to the app is actually an important evolution over the previous version. In fact, the inclusion of active images was designed with a double purpose: on the one hand, to facilitate completion of e-12HR (by tapping the images instead of directly introducing the number of standard servings consumed); and on the other to assist with estimating the quantity consumed (this new version of the app directly shows 2-4 possible options for serving sizes).

After completing the daily questionnaire with e-12HR, the information is automatically saved and sent, via 3G/4G/Wi-Fi, to the e-mail address of the research administrator (entered when first accessing the app). Once the questionnaire is completed and sent, the participant cannot change their responses or access the app until the following day.

The consumption record of the selected food groups on the app was performed for 28 consecutive days. The time interval selected is similar to other comparison/validation studies [8,12,32-34].

The questionnaire and the size of the rations used in e-12HR are based on a semiquantitative FFQ previously validated for the population of Spain [35].

**Questionnaire used in 12-hour dietary recall (e-12HR).**

1. How many pieces of fruit have you eaten today?

2. How many portions of vegetables have you eaten today?

3. How many portions of legumes (lentils, garbanzos, beans, etc) have you eaten today?

4. How many portions of chicken/turkey have you eaten today?

5. How many portions of fish have you eaten today?

6. How many portions of red meat (beef, pork, lamb) have you eaten today?

7. How many servings of soft drinks have you had today?

8. How many portions of commercially produced sweets (not home-made) (cookies/pastries) have you eaten today?

9. How many portions of prepared/frozen foods have you eaten today (croquettes, pizza, etc)?

10. How many servings of beer have you consumed today?

### Completing the Four Estimated Dietary Records

During the 28-day period that e-12HR was in use for each participant, four estimated DRs (on paper) were scheduled on randomly assigned, non-consecutive days [9,32]: three days during the weekdays and one weekend day [9,12,13,32]. The choice of between three and seven DRs is normally considered sufficient to evaluate food group intake [9,36]. Four estimated DRs were chosen instead of weighed DRs for logistical reasons [9,12]. In the daily life of the participants that made up the sample (university students who spend a large part of their days outside the home at their center of study), weighing food is not feasible and represents a great workload when compared with estimating servings consumed.

Each participant, during the first interview, received an explanation of how to use the estimated DRs and how to estimate the serving size consumed [12,13], using a pamphlet with a series of 2-4 color photographs [10,13,14,27] (one series for each food group). To assist with estimating serving sizes, each photograph was accompanied by explanatory text and three reference objects of known/predictable size for the students [30,31] (fiducial markers): a commonly used pencil, pen, and a marker.

The DRs used were based on a DR previously validated for another European country [13], but structured according to the typical Spanish diet (breakfast, lunch, an afternoon snack, and dinner), and pre-codified with a column that included the same 10 food groups selected for the e-12HR. The serving sizes were based on a semiquantitative FFQ previously validated for the Spanish population [35].

Participants were told that they must record the consumption data on a separate page for each day [7], and immediately after consuming the food [7,13].

### Second Interview: Completing the Semiquantitative Food Frequency Questionnaire

At the end of the e-12HR data collection period, each participant was scheduled for a second personal interview at their convenience. In this interview, the participant was required to fill out a structured, semiquantitative FFQ (on paper) that included the same 10 food groups selected for the e-12HR. The same research team member who performed the first interview then explained to each participant, in this second interview, the process for completing the semiquantitative FFQ, and provided them with an explanatory pamphlet to estimate what was considered a standard serving for each food group. This pamphlet contained a photograph of a standard serving for each food group along with explanatory text and three reference objects of a known/predictable size for students [30,31] (fiducial markers): a commonly used pencil, pen, and marker. The time period considered by the FFQ corresponded to the 28 days of the app. All participants completed the semiquantitative FFQ within the first week of finishing the e-12HR app with the exception of two participants, who completed the FFQ 8 and 11 days later.

The semiquantitative FFQ, as well as standard serving sizes, was based on a semiquantitative FFQ previously validated for the Spanish population [35].

Finally, in the second personal interview, participants were asked how much time, on average, was needed to complete the daily questionnaire on the app. Participants could choose between various options. Of the total participants, 11% (10/87) selected the option *less than 1 minute per day*; 36% (31/87) selected the option *approximately 1 minute per day*; 28% (24/87) chose *approximately 2 minutes per day*; 17% (15/87) chose *approximately 3 minutes per day*; 7% (6/87) chose *approximately 4 minutes per day*; and 1% (1/87) chose the *5 minutes per day or more* option. Almost half of the participants, 47% (41/87) indicated that the task took 1 minute or less per day to complete and 75% (65/87) stated that it took 2 minutes or less. For this reason, the research team considers that the time necessary to complete the app is, normally, 2 minutes per day or less.

### Legal Considerations

The study was carried out according to the rules established by the Declaration of Helsinki and the Law on Biomedical Research [37].

All of the personal information collected in this study was anonymized in accordance with current Spanish legislation [38]. To achieve this, each participant was assigned a personal alphanumeric code that they had to enter into the e-12HR app (only when first accessing the app) and include on the different paper questionnaires that were provided: the initial questionnaire (personal information), on the four estimated DRs, and on the semiquantitative FFQ. The personal alphanumeric code was used to match all of the data pertaining to the participant while at the same time respecting their anonymity.

### Data Conversion

Using the e-12HR app, each participant recorded the number of standard serving sizes consumed daily for each food group throughout the 28-day study period. With the four estimated DRs, each participant collected the number of standard serving sizes consumed daily for each food group on four different days throughout the 28-day monitoring period. On the semiquantitative FFQ, each participant selected the number of standard serving sizes habitually consumed for each food group throughout the 28-day monitoring period.

For each participant, the data from the e-12HR app, the four DRs and the FFQ had to be expressed in the same categories of habitual consumption to make comparisons: *Less than once a week; Once or twice a week, 3-4 times a week, 5-6 times a week; Once or twice a day, and 3 or more times a day*. On the FFQ, these different options for habitual consumption were already available for the participants to choose from and, as such, the FFQ data was not modified. Regarding the e-12HR app, the data needed to be transformed. As an example, one participant registered an average daily consumption of 0.52 standard servings of legumes over 28 days using the app. This average consumption represents 3.64 standard servings per week (0.52 × 7 = 3.64), which would be classified as *3–4 times per week* [25]. As for the four DRs, the information they contained also needed to be converted [9]. As an example, one participant recorded consuming 0, 0.3, and 1 standard piece of fruit on the DRs during the weekdays and 1.5 standard pieces of fruit on the DR completed at the weekend. This consumption represents an average daily consumption during weekdays of: (0 + 0.3 + 1)/3 = 0.43 standard pieces. As for weekly consumption, the conversion was as follows: (0.43 × 5 weekdays) + (1.5 × 2 weekend days) = 2.17 + 3 = 5.17 standard pieces, which would then be classified as *5-6 times per week*.

To enable making comparisons, the three tools registered the consumption of the same food groups, used the same standard servings as a reference and the intake record corresponded to the same time period, to avoid possible variations in the specific diets during different periods [20,32,39,40].

### Statistical Analysis

In this study, when comparing e-12HR with the FFQ and the four DRs for each of the selected food groups, the association between the categories of habitual consumption was evaluated using the Spearman correlation coefficient (SCC) whereas the degree of agreement between the categories of habitual consumption was evaluated using cross-classification analysis and the weighted kappa index [1,41]. For the cross-classification analysis, the percentage of participants classified in the same group was labeled *exact agreement*; in the same category or adjacent categories, *exact agreement + adjacent*; or in opposite categories, *extreme disagreement*, using different methods. For weighted kappa, partial values were assigned using the statistical program Stata: from 1.00 for an exact agreement to 0.00 for extreme disagreement.

The comparison criteria considered in this study were: average SCC≥0.5 [1,32]; average cross-classification percentage in the *exact agreement* category ≥50% [1], in the *exact agreement + adjacent* ≥75% [32], and in the *extreme disagreement* category <10%; with an average κ≥0.41 [1].

The statistical analysis was performed utilizing STATA MP 13.1 (Stata Corp LP, College Station, Texas, USA), and a *P* value < .05 was considered statistically significant [42].

## Results

### Overview

Of the 95 participants who signed the informed consent, 8 did not complete the study. Results of these individuals were not included in later statistical analyses. Participants who did not complete the study were those who performed the task fewer than 22 days, did not complete the FFQ and did not complete the four DRs.

Of the 87 participants who completed the study, 78% (68/87) completed the task every day (28 days of monitoring), 94% (82/87) of the participants completed the app at least 24 days, whereas the remaining 6% (5/87) completed the app for 22 days (Table 1).

The average age of participants was 19.2 years old; 64% (56/87) were women, and 36% were men (31/87), and 67% (58/87) were medical students and 33% (29/87) were pharmacy students (Table 1). No statistically significant differences in the variables studied were found among participants who completed the study and those who did not.

### 12-Hour Dietary Recall Versus Food Frequency Questionnaire

For all food groups: the average SCC was .70 (per food group, from .50 [legumes] to .86 [beer]; Table 2). Cross-classification analysis showed that the average percentage of individuals classified in the *exact agreement* category was 51.5% (per food group, from 37.9% [chicken/turkey] to 70.1% [beer]); *exact agreement + adjacent* was 91.8% (per food group, from 85.1% [sweets] to 96.6% [legumes & prepared foods]; Table 3); and no participants (0%) were classified in the *extreme disagreement* category. The average weighted kappa was .51 (per food group, from .34 [chicken/turkey] to .66 [fruit & beer]; Table 4).

### 12-Hour Dietary Recall Versus the Four Dietary Records

For all food groups: the average SCC was 0.63 (per food group from 0.46 [legumes] to 0.83 [fruit]; Table 2). Cross-classification analysis showed that the average percentage of individuals classified in the *exact agreement* category was 47.1% (per food group, from 31.0% [chicken/turkey] to 66.7% [beer]); *exact agreement + adjacent* was 89.2% (per food group, from 83.9% [soft drinks] to 95.4% [legumes]; Table 3); and no participants (0%) were classified in the category of *extreme disagreement*. The average weighted kappa was 0.47 (per food group, from 0.26 [fish] to 0.72 [fruit]; Table 4).

**Table 1:** Characteristics of study participants.

Characteristics		n (%)	Mean (SD)	95% CI
Participants who completed the study		87	–^a^	–
**Number of days completed the app**				
	28	68 (78.2)	–	–
	27	5 (5.7)	–	–
	26	3 (3.4)	–	–
	25	5 (5.7)	–	–
	24	1 (1.1)	–	–
	22	5 (5.7)	–	–
Age (years)		–	19.2 (3.3)	–
**Gender**				
	Female	56 (64.4)	–	74.4-53.4
	Male	31 (35.6)	–	25.6-46.6
**Faculty**				
	Medicine	58 (66.7)	–	55.7-76.4
	Pharmacy	29 (33.3)	–	44.3-23.6

^a^Not applicable.

**Table 2:** Spearman correlation coefficient (SCC) derived from 12-hour dietary recall (e-12HR) versus the food frequency questionnaire (FFQ) and from e-12HR versus the four dietary records (DRs).

Comparison	e-12HR vs FFQ		e-12HR vs DRs	
	SCC	*P* value	SCC	*P* value
Fruit	0.84	<.001	0.83	<.001
Vegetables	0.80	<.001	0.72	<.001
Legumes	0.50	<.001	0.46	<.001
Chicken/turkey	0.53	<.001	0.60	<.001
Fish	0.65	<.001	0.47	<.001
Red meat	0.69	<.001	0.50	<.001
Soft drinks	0.81	<.001	0.72	<.001
Sweets	0.71	<.001	0.74	<.001
Prepared foods	0.61	<.001	0.60	<.001
Beer	0.86	<.001	0.70	<.001
Average	0.70	N/A^a^	0.63	N/A

^a^N/A: not applicable.

**Table 3:** Cross-classification analysis derived from electronic 12-hour dietary recall (e-12HR) versus the food frequency questionnaire (FFQ) and from e-12HR versus the four dietary records (DRs).

Comparison	e-12HR vs FFQ		e-12HR vs DR		
	Exact agreement^a^ (%)	Exact agreement + adjacent^b^ (%)	Exact agreement (%)		Exact agreement + adjacent (%)
Fruit	54.0	87.4	65.5	93.1	
Vegetables	49.4	92.0	39.1	90.8	
Legumes	56.3	96.6	55.2	95.4	
Chicken/turkey	37.9	86.2	31.0	87.4	
Fish	48.3	90.8	32.2	88.5	
Red meat	58.6	95.4	39.1	88.5	
Soft drinks	52.9	95.4	57.5	83.9	
Sweets	40.2	85.1	40.2	89.6	
Prepared foods	47.1	96.6	44.8	88.5	
Beer	70.1	92.0	66.7	86.2	
Average	51.5	91.8	47.1	89.2	

^a^Exact agreement: % of cases cross-classified into the same category.

^b^Exact agreement + adjacent: % of cases cross-classified into the same or adjacent category.

**Table 4:** Weighted kappa derived from electronic 12-hour dietary recall (e-12HR) versus the food frequency questionnaire (FFQ) and from e-12HR versus the four dietary records (DRs).

Comparison	e-12HR vs FFQ		e-12HR vs DRs	
	Kappa	*P* value	Kappa	*P* value
Fruit	0.66	<.001	0.72	<.001
Vegetables	0.61	<.001	0.51	<.001
Legumes	0.38	<.001	0.34	<.001
Chicken/turkey	0.34	<.001	0.36	<.001
Fish	0.41	<.001	0.26	<.001
Red meat	0.54	<.001	0.34	<.001
Soft drinks	0.59	<.001	0.60	<.001
Sweets	0.48	<.001	0.49	<.001
Prepared foods	0.41	<.001	0.44	<.001
Beer	0.66	<.001	0.63	<.001
Average	0.51	N/A^a^	0.47	N/A

^a^N/A: not applicable.

## Discussion

### Overview

Current-day recall is a modified 24-hour recall focused on a series of food groups and completed at the end of every day during the monitoring period. This method has been designed to classify individuals according to categories of habitual consumption of selected food groups and is not intended to be used to determine total food consumption for an individual [25]. This study is the first time that current-day recall, based on the e-12HR app, has been compared with two different reference models, one long-term (the FFQ) and the other short-term (four DRs) to determine the utility of this new method as a tool for estimating the usual dietary intake of a series of food groups.

### Reference Methods

Evaluating the *true validity* of a method requires measuring, with a high degree of accuracy, the habitual diet of free-living individuals during a prolonged period of time, which is not feasible [1]. As a result, the researchers of this study have evaluated the *relative validity* of the method by comparing it with an alternative method of dietary assessment, with its own limitations [1]. Given that none of the comparison methods are perfect [5,8,39,43], as all are subjective, it is of crucial importance for their errors to be as independent as possible to avoid falsely attributing validity to the method in question [5,6,8,16,20,34,44]. For this reason, the research team considered the FFQ and DRs to be possible reference methods and not 24-hour recalls (current-day recall is a modified 24-hour recall). As neither the FFQ nor the DRs can be considered definitive reference methods, the research team selected both methods for this validation study. Aware that the quantity of work for participants could be excessive, a semiquantitative FFQ was selected (to be completed at the end of the e-12HR monitoring period) and four DRs (estimated).

### Principal Results

#### 12-Hour Dietary Recall Versus the Food Frequency Questionnaire

For all food groups: the average SCC was 0.70 (in 10/10 food groups SCC≥0.50 [1,32]). Cross-classification analysis showed that the average percentage of individuals classified in the category of *exact agreement* was 51.5% (in 5/10 food groups ≥50% [1]); *exact agreement + adjacent* was 91.8% (10/10 food groups ≥75% [32]); and no participants (0%) were classified in the category of *extreme disagreement* [1]. The average weighted kappa was .51 (in 8/10 food groups κ≥0.41 [1]). These values indicate good agreement between the two methods [1,32]). Values obtained were very similar to those from previous studies with the older version of the e-12HR app, which did not use photographs to facilitate estimation of servings consumed [45].

#### 12-Hour Dietary Recall Versus the Four Dietary Records

For all food groups: the average SCC was 0.63 (in 8/10 food groups SCC≥0.50 [1,32]). Cross-classification analysis showed that the average percentage of individuals classified in the category of *exact agreement* was 47.1% (in 4/10 food groups ≥50% [1]); *exact agreement + adjacent* was 89.2% (10/10 food groups ≥75% [32]); and no participants (0%) were classified in the *extreme disagreement* category [1]. The average weighted kappa was 0.47 (in 6/10 food groups κ≥0.41 [1]). These values indicate good agreement between the two methods [1,32]). This is the first time that the e-12HR app has been compared with the DRs and as such there are no prior results for reference.

It is important to note that the cross-classification analysis and k depend on the number of categories used [1]. With the goal of reducing this dependence when evaluating the degree of agreement between different methods, the six original categories could have been reorganized into three [1,8,25], four [32,33] or five [46,47], as other authors have done. Notwithstanding, this research team preferred to maintain six categories for the statistical analysis [45] as a greater number of categories of habitual consumption provides compact information on the ability of methods to assign individuals according to agreement with the distribution of dietary intake [46]. Comparison criteria applied to this study were also considered by Masson et al [1] and Forster et al [32]. However, considering that these authors only used three and four categories of habitual consumption, respectively, the demands of this study are much more restrictive. In this study, the classification of the *exact agreement* categories *suffered* especially, whereas the criteria used by Mason et al (*exact agreement* ≥50%) was defined by three categories versus the six considered here.

The results demonstrate good agreement between e-12HR and both reference methods (FFQ and DRs). Nevertheless, certain disagreement was observed with the app. Regarding the FFQ: the average cross-classification analysis showed that 8.2% of participants were classified at a distance of 2–4 categories. As for the DRs: the average cross-classification analysis showed that 10.8% of participants were classified at a distance of 2-4 categories. The three methods (e-12HR, FFQ, and DRs) are all based on self-administered and anonymized questionnaires and use the same color photographs for directly estimating servings. Because these questionnaires were both self-administered and anonymized (with the use of alphanumeric codes), they enabled researchers to avoid information biases, such as obsequiousness bias and researcher bias in all three methods. Using the same photographs also favors the same precision in the three methods when estimating servings. In this sense, each participant serves as their own *control*, thereby minimizing differences when considering servings consumed. In any case, consumption frequency seems to have a greater impact on dietary intake than on serving size [5,9].

However, differences between the three methods are factors that could potentially contribute to the discrepancies observed. The fundamental differences between the methods are centered on both the regularity in collecting data on dietary intake and the format used in the questionnaires.

#### Regularity in Collecting Data on Dietary Intake

With DRs, the collection of data was performed after consuming each food group throughout the four days selected; with the e-12HR app, data collection takes place at the end of each day during the 28-day monitoring period; in the FFQ, data was collected at the end of the monitoring period. These differences in regularity mean that the accuracy of the information depends on both the memory of the participant and the daily variation in dietary intake among free-living individuals.

With DRs, dependance on memory is not an issue [12]; the e-12HR app is short-term memory dependent; while FFQs depend on long-term memory.

The e-12HR does not interfere with daily variation as it is completed every day; however, an effect is seen on the FFQ, with only one point of data collected, and the DRs, where data is collected sporadically (only 4/28 days during the monitoring period).

#### Format Used in Questionnaires

The app is digital and both the FFQ and DRs are completed on paper. Paper formats are typically associated with errors such as unanswered questions, questions with multiple responses [10] (FFQ), and not registering the quantity consumed for some of the different food groups selected (DRs) [48]. In addition, the information on paper questionnaires must be converted subsequently to digital format for analysis, which increases both the workload and costs for the research team [17,40]. Online FFQs and DRs, or even smartphone apps, offer direct solutions to these limitations [10,13,20,30-32,40,49-53], by reducing paper waste and mailing costs, and removing the need for secure storage space and organization inherent with paper documents [50]. Additionally, online administrative methods can be used to gain access to specific population groups that would otherwise be difficult to study due to geography, for example, as they can be used remotely [32]. Despite the potential advantages of utilizing FFQs and DRs in digital format, in the end it was decided to use paper formats in this study. The research team took into account that evidence shows that data collected from smartphone apps and both Web-based FFQs and DRs are comparable with data from paper formats [7,14,16,27,29,31-34,40,46,54]. The team also considered the characteristics of this study and the potential disadvantages of developing FFQs and DRs in a digital format that could outweigh the possible benefits. In fact, in this study, the paper-based FFQ and the DRs were very short and simple (they only contain 10 food groups), and the sample population is made up of students at the Schools of Medicine and Pharmacy at the University of Seville, which was easily accessible for the research team.

The simplicity of the paper-based FFQ and DRs minimized possible errors, the amount of paper used, problems with storage space, and costs associated with data conversion. These costs were minimal when compared with the potential costs of developing an online or smartphone-based FFQ and DRs.

Easy access to the sample made it possible to complete the paper FFQ in person, without the need for researchers or participants to travel or pay mailing costs [25].

### Strengths and Limitations

The strengths of this study include that the e-12HR is a tool that depends only on short-term memory (the app is completed at the end of each day), it does not interfere with variability of dietary intake in daily life (the app is completed daily). In addition, it has a digital format (young people and adolescents have shown their preference for methods that use digital technology versus traditional methods [31,53]—improving motivation and completion rates [48]). These characteristics support greater accuracy in assigning the category of habitual consumption in those cases where the ranking assigned by current-day recall is different from that assigned by reference methods. Also, with the e-12HR app, data collection is performed digitally, eliminating the need for investigators to enter the data manually; it is a self-reporting tool, not requiring interviewers; it is both simple and intuitive to use, with photographs to assist with estimating servings consumed; and overall research costs are greatly reduced.

Limitations of this study include that the sample used was extremely educated, which is a convenient sample and not representative of the youth population on the national level. Another limitation derives from the need to have a smartphone with an Android operating system. Access to these technologies is not universal and could exclude those students with less purchasing power [25]. Also, even though two different reference methods were used for the e-12HR app, it is important to note that the high degree of association and agreement between the data collected when comparing different methods does not indicate that current-day recall is exact, as there is no true measurement of dietary intake [5,8,39,43]. Ideally, validation studies should include the use of nutritional biomarkers, but currently there are few biomarkers for specific foods [12,43,44,55] and they cannot measure habitual intake [43].

### Future Research

Future research will focus on the selection of other study samples, additional food groups, different monitoring periods for comparison, and with varying regularity for completing the app from daily, as in this study, to two days, three days, etc.

### Conclusions

The good agreement between e-12HR and both reference methods, as illustrated by the results of various statistical analyses demonstrate the *relative validity* of current-day recall for determining categories of habitual intake for specific food groups, and even for those foods that are consumed infrequently. For large-scale epidemiological studies with samples of individuals who have grown up using new technologies and that do not require determining the complete diet, e-12HR can be a useful tool to replace other methods which are oftentimes longer, more expensive, and require a larger workload for both participants and researchers. Additionally, e-12HR can be a useful tool in a clinical context. Healthcare professionals can use the app with patients/users who are accustomed to new technologies. Individual information obtained through the app could be used as a baseline for health education and promotion activities: specific activities for each patient/user focused on those specific food groups that require a change in consumption patterns.

## Abbreviations

DR: dietary record
e-12HR: electronic 12-hour dietary recall
FFQ: food frequency questionnaire

## References

[1] Masson LF, McNeill G, Tomany JO, Simpson JA, Peace HS, Wei L, Grubb DA, Bolton-Smith C (2003). Statistical approaches for assessing the relative validity of a food-frequency questionnaire: use of correlation coefficients and the kappa statistic. Public Health Nutr.

[2] Giabbanelli PJ, Adams J (2016). Identifying small groups of foods that can predict achievement of key dietary recommendations: data mining of the UK National Diet and Nutrition Survey, 2008-12. Public Health Nutr.

[3] Branscum P, Sharma M, Kaye G, Succop P (2010). An evaluation of the validity and reliability of a food behavior checklist modified for children. J Nutr Educ Behav.

[4] Carroll RJ, Midthune D, Subar AF, Shumakovich M, Freedman LS, Thompson FE, Kipnis V (2012). Taking advantage of the strengths of 2 different dietary assessment instruments to improve intake estimates for nutritional epidemiology. Am J Epidemiol.

[5] Willett W (2013). Nutritional Epidemiology. 3rd edition.

[6] Stumbo PJ (2013). New technology in dietary assessment: a review of digital methods in improving food record accuracy. Proc Nutr Soc.

[7] Saloheimo T, González SA, Erkkola M, Milauskas DM, Meisel JD, Champagne CM, Tudor-Locke C, Sarmiento O, Katzmarzyk PT, Fogelholm M (2015). The reliability and validity of a short food frequency questionnaire among 9-11-year olds: a multinational study on three middle-income and high-income countries. Int J Obes Suppl.

[8] Cleghorn CL, Harrison RA, Ransley JK, Wilkinson S, Thomas J, Cade JE (2016). Can a dietary quality score derived from a short-form FFQ assess dietary quality in UK adult population surveys?. Public Health Nutr.

[9] Saeedi P, Skeaff SA, Wong JE, Skidmore PML (2016). Reproducibility and Relative Validity of a Short Food Frequency Questionnaire in 9-10 Year-Old Children. Nutrients.

[10] Kristal AR, Kolar AS, Fisher JL, Plascak JJ, Stumbo PJ, Weiss R, Paskett ED (2014). Evaluation of web-based, self-administered, graphical food frequency questionnaire. J Acad Nutr Diet.

[11] Rutishauser IHE (2005). Dietary intake measurements. Public Health Nutr.

[12] Buch-Andersen T, Pérez-Cueto FJA, Toft U (2016). Relative validity and reproducibility of a parent-administered semi-quantitative FFQ for assessing food intake in Danish children aged 3-9 years. Public Health Nutr.

[13] Knudsen VK, Hatch EE, Cueto H, Tucker KL, Wise L, Christensen T, Mikkelsen EM (2016). Relative validity of a semi-quantitative, web-based FFQ used in the 'Snart Forældre' cohort - a Danish study of diet and fertility. Public Health Nutr.

[14] Medin AC, Carlsen MH, Andersen LF (2016). Associations between reported intakes of carotenoid-rich foods and concentrations of carotenoids in plasma: a validation study of a web-based food recall for children and adolescents. Public Health Nutr.

[15] Gibson R (2005). Principles of Nutritional Assessment, 2nd edition.

[16] Tucker KL, Smith CE, Lai C, Ordovas JM (2013). Quantifying diet for nutrigenomic studies. Annu Rev Nutr.

[17] Martín-Moreno JM, Gorgojo L (2007). [Assessment of dietary intake at the population level through individual questionnaires: methodological shadows and lights]. Rev Esp Salud Publica.

[18] Dhurandhar NV, Schoeller D, Brown AW, Heymsfield SB, Thomas D, Sørensen TIA, Speakman JR, Jeansonne M, Allison DB, the EBMWG (2014). Energy balance measurement: when something is not better than nothing. Int J Obes (Lond).

[19] Shamah-Levy T, Rodríguez-Ramírez S, Gaona-Pineda EB, Cuevas-Nasu L, Carriquiry AL, Rivera JA (2016). Three 24-Hour Recalls in Comparison with One Improve the Estimates of Energy and Nutrient Intakes in an Urban Mexican Population. J Nutr.

[20] Svensson A, Larsson C (2015). A Mobile Phone App for Dietary Intake Assessment in Adolescents: An Evaluation Study. JMIR Mhealth Uhealth.

[21] Illner A, Freisling H, Boeing H, Huybrechts I, Crispim SP, Slimani N (2012). Review and evaluation of innovative technologies for measuring diet in nutritional epidemiology. Int J Epidemiol.

[22] Steele R (2015). An overview of the state of the art of automated capture of dietary intake information. Crit Rev Food Sci Nutr.

[23] Hassannejad H, Matrella G, Ciampolini P, De MI, Mordonini M, Cagnoni S (2017). Automatic diet monitoring: a review of computer vision and wearable sensor-based methods. Int J Food Sci Nutr.

[24] Yoke JT, Thuong N, Qing Z, Karunanithi M, Lei Y (2017). Predicting food nutrition facts using pocket-size near-infrared sensor. Conf Proc IEEE Eng Med Biol Soc.

[25] Bejar LM, Sharp BN, García-Perea MD (2016). The e-EPIDEMIOLOGY Mobile Phone App for Dietary Intake Assessment: Comparison with a Food Frequency Questionnaire. JMIR Res Protoc.

[26] Souverein OW, Dekkers AL, Geelen A, Haubrock J, de VJH, Ocké MC, Harttig U, Boeing H, van 'VP (2011). Comparing four methods to estimate usual intake distributions. Eur J Clin Nutr.

[27] Medin AC, Astrup H, Kåsin BM, Andersen LF (2015). Evaluation of a Web-Based Food Record for Children Using Direct Unobtrusive Lunch Observations: A Validation Study. J Med Internet Res.

[28] Gerdessen JC, Souverein OW, van 't Veern P, de Vries JH (2015). Optimising the selection of food items for FFQs using Mixed Integer Linear Programming. Public Health Nutr.

[29] Henriksson H, Bonn SE, Bergström A, Bälter K, Bälter O, Delisle C, Forsum E, Löf M (2015). A new mobile phone-based tool for assessing energy and certain food intakes in young children: a validation study. JMIR Mhealth Uhealth.

[30] Daugherty BL, Schap TE, Ettienne-Gittens R, Zhu FM, Bosch M, Delp EJ, Ebert DS, Kerr DA, Boushey CJ (2012). Novel technologies for assessing dietary intake: evaluating the usability of a mobile telephone food record among adults and adolescents. J Med Internet Res.

[31] Casperson SL, Sieling J, Moon J, Johnson L, Roemmich JN, Whigham L (2015). A mobile phone food record app to digitally capture dietary intake for adolescents in a free-living environment: usability study. JMIR Mhealth Uhealth.

[32] Forster H, Fallaize R, Gallagher C, O'Donovan CB, Woolhead C, Walsh MC, Macready AL, Lovegrove JA, Mathers JC, Gibney MJ, Brennan L, Gibney ER (2014). Online dietary intake estimation: the Food4Me food frequency questionnaire. J Med Internet Res.

[33] Fallaize R, Forster H, Macready AL, Walsh MC, Mathers JC, Brennan L, Gibney ER, Gibney MJ, Lovegrove JA (2014). Online dietary intake estimation: reproducibility and validity of the Food4Me food frequency questionnaire against a 4-day weighed food record. J Med Internet Res.

[34] Marshall SJ, Livingstone KM, Celis-Morales C, Forster H, Fallaize R, O'Donovan CB, Woolhead C, Marsaux CF, Macready AL, Navas-Carretero S, San-Cristobal R, Kolossa S, Tsirigoti L, Lambrinou CP, Moschonis G, Godlewska M, Surwiłło A, Drevon CA, Manios Y, Traczyk I, Martínez JA, Saris WH, Daniel H, Gibney ER, Brennan L, Walsh MC, Lovegrove JA, Gibney M, Mathers JC, Food4Me Study (2016). Reproducibility of the Online Food4Me Food-Frequency Questionnaire for Estimating Dietary Intakes across Europe. J Nutr.

[35] Rodríguez IT, Ballart JF, Pastor GC, Jordà EB, Val VA (2008). [Validation of a short questionnaire on frequency of dietary intake: reproducibility and validity]. Nutr Hosp.

[36] Vereecken CA, Maes L (2003). A Belgian study on the reliability and relative validity of the Health Behaviour in School-Aged Children food-frequency questionnaire. Public Health Nutr.

[37] (2007). Head of State.

[38] (1999). Head of State.

[39] Watanabe M, Yamaoka K, Yokotsuka M, Adachi M, Tango T (2011). Validity and reproducibility of the FFQ (FFQW82) for dietary assessment in female adolescents. Public Health Nutr.

[40] Rangan AM, O'Connor S, Giannelli V, Yap ML, Tang LM, Roy R, Louie JCY, Hebden L, Kay J, Allman-Farinelli M (2015). Electronic Dietary Intake Assessment (e-DIA): Comparison of a Mobile Phone Digital Entry App for Dietary Data Collection With 24-Hour Dietary Recalls. JMIR Mhealth Uhealth.

[41] Viera AJ, Garrett JM (2005). Understanding interobserver agreement: the kappa statistic. Fam Med.

[42] StataCorp L 1.

[43] England CY, Thompson JL, Jago R, Cooper AR, Andrews RC (2017). Development of a brief, reliable and valid diet assessment tool for impaired glucose tolerance and diabetes: the UK Diabetes and Diet Questionnaire. Public Health Nutr.

[44] Bassett JK, English DR, Fahey MT, Forbes AB, Gurrin LC, Simpson JA, Brinkman MT, Giles GG, Hodge AM (2016). Validity and calibration of the FFQ used in the Melbourne Collaborative Cohort Study. Public Health Nutr.

[45] Béjar LM (2017). First evaluation steps of a new method for dietary intake estimation regarding a list of key food groups in adults and in different sociodemographic and health-related behaviour strata. Public Health Nutr.

[46] Tabacchi G, Filippi AR, Breda J, Censi L, Amodio E, Napoli G, Bianco A, Jemni M, Firenze A, Mammina C (2015). Comparative validity of the ASSO-Food Frequency Questionnaire for the web-based assessment of food and nutrients intake in adolescents. Food Nutr Res.

[47] Tabacchi G, Filippi AR, Amodio E, Jemni M, Bianco A, Firenze A, Mammina C (2016). A meta-analysis of the validity of FFQ targeted to adolescents. Public Health Nutr.

[48] Vereecken CA, Rossi S, Giacchi MV, Maes L (2008). Comparison of a short food-frequency questionnaire and derived indices with a seven-day diet record in Belgian and Italian children. Int J Public Health.

[49] Swierk M, Williams PG, Wilcox J, Russell KG, Meyer BJ (2011). Validation of an Australian electronic food frequency questionnaire to measure polyunsaturated fatty acid intake. Nutrition.

[50] Falomir Z, Arregui M, Madueño F, Corella D, Coltell O (2012). Automation of Food Questionnaires in Medical Studies: a state-of-the-art review and future prospects. Comput Biol Med.

[51] Labonté M, Cyr A, Baril-Gravel L, Royer M, Lamarche B (2012). Validity and reproducibility of a web-based, self-administered food frequency questionnaire. Eur J Clin Nutr.

[52] Christensen SE, Möller E, Bonn SE, Ploner A, Wright A, Sjölander A, Bälter O, Lissner L, Bälter K (2013). Two new meal- and web-based interactive food frequency questionnaires: validation of energy and macronutrient intake. J Med Internet Res.

[53] Hongu N, Pope BT, Bilgiç P, Orr BJ, Suzuki A, Kim AS, Merchant NC, Roe DJ (2015). Usability of a smartphone food picture app for assisting 24-hour dietary recall: a pilot study. Nutr Res Pract.

[54] Matthys C, Pynaert I, De KW, De HS (2007). Validity and reproducibility of an adolescent web-based food frequency questionnaire. J Am Diet Assoc.

[55] Hedrick VE, Dietrich AM, Estabrooks PA, Savla J, Serrano E, Davy BM (2012). Dietary biomarkers: advances, limitations and future directions. Nutr J.

